# Comparison of the effects of pulmonary rehabilitation with chest physical therapy on the levels of fibrinogen and albumin in patients with lung cancer awaiting lung resection: a randomized clinical trial

**DOI:** 10.1186/1471-2466-14-121

**Published:** 2014-07-28

**Authors:** Maria Tereza Aguiar Pessoa Morano, Rafael Mesquita, Guilherme Pinheiro Ferreira Da Silva, Amanda Souza Araújo, Juliana Maria De Sousa Pinto, Antero Gomes Neto, Cyntia Maria Sampaio Viana, Manoel Odorico De Moraes Filho, Eanes Delgado Barros Pereira

**Affiliations:** 1 Universidade Federal do Ceará – UFC, Fortaleza, Brazil; 2 Hospital de Messejana Dr. Carlos Alberto Studart Gomes, Fortaleza, Brazil; 3 Center of Expertise for Chronic Organ Failure (CIRO+), Maastricht University Medical Center, Horn, The Netherlands; 4 Universidade de Fortaleza – UNIFOR, Fortaleza, Brazil

**Keywords:** Non-small cell lung cancer, Inflammatory lung disease, Physical therapy, Rehabilitation, Serum albumin, Serum fibrinogen

## Abstract

**Background:**

Systemic inflammation plays an important role in the initiation, promotion, and progression of lung carcinogenesis. In patients with non-small cell lung cancer (NSCLC), fibrinogen levels correlate with neoplasia. Here we compared the effects of pulmonary rehabilitation (PR) with chest physical therapy (CPT) on fibrinogen and albumin levels in patients with LC and previous inflammatory lung disease awaiting lung resection.

**Methods:**

We conducted a randomized clinical trial with 24 patients who were randomly assigned to Pulmonary Rehabilitation (PR) and Chest Physical Therapy (CPT) groups. Each group underwent training 5 days weekly for 4 weeks. All patients were assessed before and after four weeks of training through clinical assessment, measurement of fibrinogen and albumin levels, spirometry, 6-minute Walk Test (6MWT), quality of life survey, and anxiety and depression scale. PR involved strength and endurance training, and CPT involved lung expansion techniques. Both groups attended educational classes.

**Results:**

A mixed between-within subjects analysis of variance (ANOVA) revealed a significant interaction between time (before and after intervention) and group (PR vs. CPT) on fibrinogen levels (F(1, 22) = 0.57, p < 0.0001) and a significant main effect of time (F(1, 22) = 0.68, p = 0.004). Changes in albumin levels were not statistically significant relative to the interaction effect between time and group (F(1, 22) = 0.96, p = 0.37) nor the main effects of time (F(1, 22) = 1.00, p = 1.00) and group (F(1, 22 ) = 0.59, p = 0.45). A mixed between-within subjects ANOVA revealed significant interaction effects between time and group for the peak work rate of the unsupported upper limb exercise (F(1, 22) = 0.77, p = 0.02), endurance time (F(1, 22) = 0.60, p = 0.001), levels of anxiety (F(1, 22) = 0.60, p = 0.002) and depression (F(1, 22) = 0.74, p = 0.02), and the SF-36 physical component summary (F(1, 22) = 0.83, p = 0.07).

**Conclusion:**

PR reduced serum fibrinogen levels, improved functional parameters, and quality of life of patients with LC and inflammatory lung disease awaiting lung resection.

**Trial registration:**

Current Controlled Trials RBR-3nm5bv.

## Background

Malignant neoplasms and chronic degenerative diseases replace infectious and parasitic pathologies as the main causes of death in Brazil as well as worldwide, particularly in the United States and Europe [1]. Lung cancer (LC) is one of the most common malignant neoplasms, the number of cases continues to increase by 3% annually [2], and it will become the fifth most frequently diagnosed cancer in Brazilian [3].

Surgery without adjuvant treatment is most frequently indicated to treat patients with early stages (I and II) of non-small cell lung cancer (NSCLC) [4], and surgery for patients with up to stage III may be more beneficial compared with other modalities [1,4]. Systemic inflammation plays an important role in the initiation, promotion, and progression of lung carcinogenesis [5]. In patients with NSCLC there is a relationship between fibrinogen levels and neoplasia. This is because >50% of patients with primary tumors and 90% of those with metastases present with altered coagulation—a prothrombotic state caused by the ability of cancer cells to activate the coagulation system [5-7]. Moreover, serum fibrinogen and albumin levels serve as prognostic factors for postoperative complications after lung resection [7,8].

Few studies assessed the impact of pulmonary rehabilitation on postoperative complications of patients who undergo lung resection for lung cancer [9-11], One of the postoperative outcomes observed by Morano et al. [11] was that in the PR group, fewer patients developed bronchopleural fistulas and required a chest tube for a fewer number of days. However, none of these studies used a randomized design to assess the influence of pulmonary rehabilitation on the serum levels of fibrinogen and albumin. Therefore, to address this important issue, we assessed the immediate effects of PR versus CPT on serum levels of fibrinogen and albumin in patients with NSCLC awaiting lung resection.

## Methods

### Study design and patients

We conducted a randomized clinical trial in two teaching hospitals in Fortaleza, Northeastern Brazil. The study included patients with NSCLC who were eligible for lung resection and experienced previous pulmonary disease with impaired respiratory function identified using spirometry. After selection, six groups of four patients each were randomly assigned (individual allocations were placed using sealed envelopes) to either the Pulmonary Rehabilitation (PR, three groups) or the Chest Physical Therapy (CPT, three groups) groups. Training was conducted five times weekly for 4 weeks. After receiving approval from the Ethics Committee of the Hospital de Messejana (Opinion No. 540/80), all subjects granted their informed written consent.

### Enrollment

**Figure 1:**
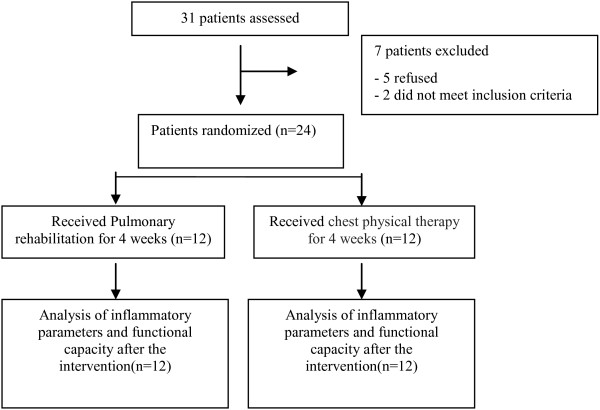
Flow diagram of the randomized clinical trial of a 4-week pulmonary rehabilitation versus chest physical therapy before lung cancer resection.

Thirty-one patients were recruited between March 2008 and March 2011, and seven patients were excluded, because five refused to participate and two did not meet inclusion criteria because of normal pulmonary function. Twenty-four patients were randomly assigned (12 each) to the PR and 12 CPT groups (Figure 1).

### Characterization of patients

Before and after training, all participants were assessed demographically and clinically (sex, age, weight, height, Body Mass Index, tobacco smoking, comorbidities, and lung cancer staging), as well as by laboratory analysis of serum levels of fibrinogen and albumin and by pulmonary function tests. Physical capacity, quality of life, levels of anxiety, and depression were also assessed. Serum levels of fibrinogen and albumin were measured using a blood sample collected in disposable Vacutainer tubes [12,13]. Pulmonary function was assessed using spirometry following international guidelines [14] with reference values for the Brazilian population [15]. Normal lung function was defined using the spirometric parameters as follows: Ratio of forced expiratory volume (FEV_1_) to forced vital capacity (FVC) ratio >0.70 and FVC and FEV_1_ > 80% of the predicted value. Physical capacity was measured using the following tests: unsupported upper limb exercise test (UULEX), endurance testing, and the 6-min walk test (6MWT). The 6MWT was performed twice over a 30-m corridor according to the American Thoracic Society (ATS) guidelines [16,17]. Quality of life was assessed using the Medical Outcomes Study 36 - Item Short Form Health Survey (SF-36) [18], which comprises 36 items divided into eight domains. The SF-36 results were grouped for the development of the Physical Component Summary (PCS) and the Mental Component Summary (MCS). The levels of anxiety and depression were determined using the Hospital Anxiety and Depression Scale (HADS) [19], which asks 14 questions divided into anxiety (HDAS-A) and depression (HADS-D) scales.

### Interventions

The group of patients undergoing PR was submitted to upper limb (UL) and lower limb (LL) stretching exercises, warm-up exercises, UL strengthening exercises, aerobic conditioning, and inspiratory muscle training. UL strengthening started at 50% of peak work capacity during preliminary testing, and it was performed using Proprioceptive Neuromuscular Facilitation (PNF) diagonal movements with barbells. Aerobic conditioning was performed on a treadmill at 80% of peak work capacity determined by endurance testing. The group of patients who underwent CPT followed the routine protocol of the hospital comprising lung expansion techniques (sustained maximum inspiration, fractional inspiration, breathing patterns, pursed lip breathing, and use of a flow-based incentive spirometer (Respiron®, NCS, Brazil). Both groups attended educational sessions informing them of the importance of pre- and postoperative care, the surgical process, energy conservation techniques, as well as relaxation, stress, and nutritional management techniques.

### Statistical analysis

Data analysis was performed using the Statistical Package for the Social Sciences 13.0 and GraphPad Prism 5.0. The Kolmogorov-Smirnov test was used to test for normality. Data are presented as absolute or relative frequency, or both, represented by the mean ± standard deviation of normally distributed data or median (interquartile interval) for data not normally distributed. The variation (delta) between pre- and postoperative periods is presented as the mean (confidence interval, CI 95%). The Mann–Whitney U test was used to compare continuous variables between the two groups at baseline. The values of continuous variables as a function of time were compared between groups using a mixed between-within subjects ANOVA. P <0.05 indicates statistical significance.

## Results

All 24 participants successfully completed the training assignments. Nineteen patients were diagnosed with chronic obstructive pulmonary disease (COPD), two with interstitial lung disease, and three with bronchiectasis. Their characteristics are presented in Table 1.

**Table 1:** Baseline characteristics of the participants

**Variable**	**PR (n = 12)**	**CPT (n = 12)**	***P***
Sex (M/F)*	4/8	5/7	0.46
**Age (years)mean ± standard deviation†**	65 ± 8	69 ± 7	0.33
**BMI (kg · m**^**-2**^**) mean ± standard deviation**	26 ± 5	24 ± 6	0.56
Current smoker (n/%)*	10/83	9/75	0.44
Comorbidity (n/%)*	7/58	4/33	0.35
With COPD (n/%)*	9/75	10/83	0.62
LC stage (n/%)*			
**Stage I/II**	11/92	9/75	0.16
**Stage III A**	1/8	3/25	
**Lung function**			
**FEV**_**1 **_**(%)mean ± standard deviation†**	48 **±** 14	49 ± 12	0.41
**FVC (%)median(25%-75%)‡**	63 (53 – 93)	63 (50 – 71)	0.26
**FEV**_**1**_**/FVC (%)median(25%-75%)‡**	62 (51 – 73)	56 (53 – 61)	0.71
Arterial blood gas			
PaCO_2_	41.8 ± 3.8	42.5 ± 3.6	0.3
**PaO**_**2**_	75.8 ± 5.8	79.9 ± 1.8	0.2
**Fibrinogen (mg/dl) median (25%-75%)‡**	414 (366 – 588)	368 (318 – 527)	0.31
**Albumin (g/dl) mean ± standard deviation†**	4.1 ± 0.6	3.9 ± 0.3	0.35
**Physical capacity**			
**UULEX (kg)median (25%-75%)‡**	1 (1 – 1.5)	0.75 (0.5 – 1)	0.08
**Endurance testing(s) mean ± standard deviation†**	429 ± 156	503 ± 216	0.35
**6MWT(m) mean ± standard deviation†**	425 ± 85	339 ± 108	0.06
**Quality of life**			
**SF-36 PCS (%)mean ± standard deviation†**	36 ± 8	36 ± 8	0.99
**SF-36 MCS (%)mean ± standard deviation†**	44 ± 13	43 ± 13	0.86
**Anxiety and Depression**			
***HADS-A *** **(scores)median (25%-75%)‡**	12 (6 – 13)	10 (4 – 14)	0.72
***HADS-D *** **(scores) mean ± standard deviation†**	10 ± 5	10 ± 3	0.75

*Fisher exat test. †student t test ‡Mann–Whitney U test. PR: Pulmonary Rehabilitation group; CPT: Chest Physical Therapy group; M: Male; F: Female; BMI: Body Mass Index; COPD: Chronic Obstructive Pulmonary Disease; LC: Lung Cancer; PaO_2_: Partial pressure of Oxygen in arterial blood; PaCO_2_: Partial pressure of Carbon Dioxide in arterial blood; FEV_1_: Forced Expiratory Volume at the first second; FVC: Forced Vital Capacity; UULEX: unsupported upper limb exercise test; 6MWT: 6-minute Walk Test; PCS: SF-36 Physical Component Summary; MCS: SF-36 Mental Component Summary; *HADS-A*: Hospital Anxiety and Depression Scale – Anxiety; *HADS-D*: Hospital Anxiety and Depression Scale – Depression. Student’s t-test was used for continuous variables and Chi-squared test was used for categorical variables.

**Figure 2:**
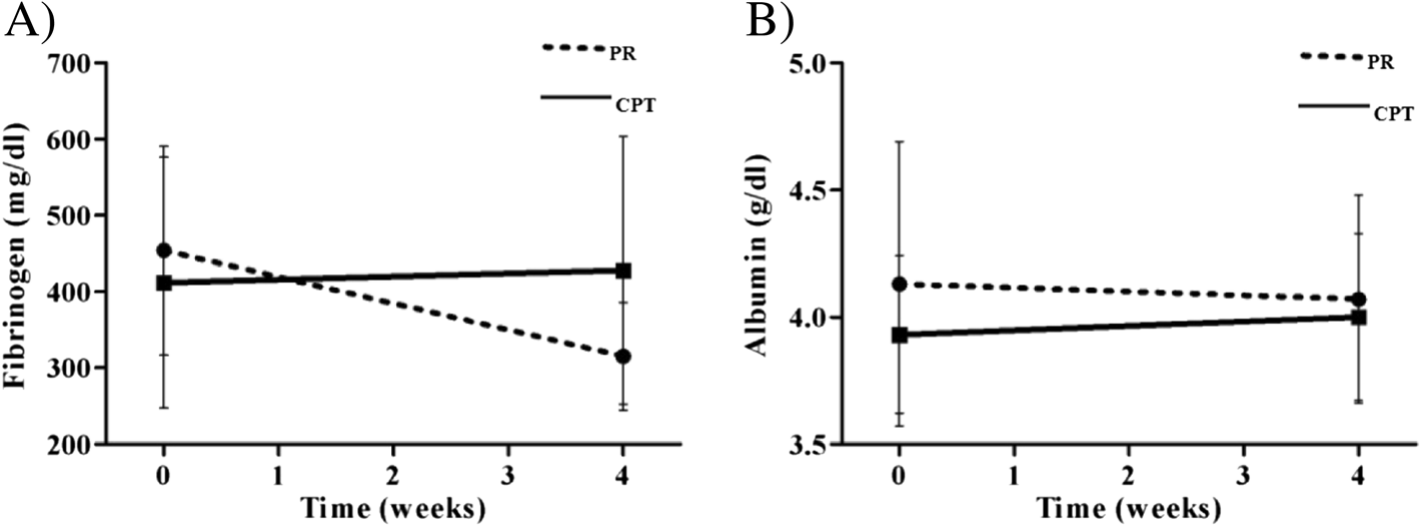
**Comparison of serum fibrinogen and albumin levels between pre- and post-intervention in the PR and CPT groups (A and B).** PR group: dotted line; CPT group: continuous line.

Both groups comprised mostly women, older people, smokers, and patients with Stage I or II lung cancer. The two groups were equivalent regarding the presence of COPD and severity of the disease. Of the patients in the PR and CPT groups, 50% and 60% were classified as Stage III of the Global Initiative for Chronic Obstructive Lung Disease, respectively (p = 0.1). The groups did not differ systematically at the outset.Fibrinogen levels before and after each intervention are presented in Figure 2A. A mixed between-within subjects ANOVA revealed a statistically significant interaction effect between time (before and after intervention) and group (PR vs. CPT) (F(1, 22) = 0.57, p < 0.0001) and a statistically significant main effect for time (F(1, 22) = 0.68, p = 0.004). The main effect for group was not statistically significant (F(1, 22) = 0.41, p = 0.53). Albumin levels before and after each intervention are presented in Figure 2B. Neither the interaction effect between time and group (F(1, 22) = 0.96, p = 0.37) nor the main effects of time (F(1, 22) = 1.00, p = 1.00) and group (F(1, 22) = 0.59, p = 0.45) was statistically significant.

Table 2 presents baseline and 1-month physical capacity, quality of life, anxiety, and depression for each group. A mixed between-within subjects ANOVA revealed statistically significant interaction effects between time and group for the peak work rate of the UULEX (F(1, 22) = 0.77, p = 0.02), endurance time (F(1, 22) = 0.60, p = 0.001), and the levels of anxiety (F(1, 22) = 0.60, p = 0.002) and depression (F(1, 22) = 0.74, p = 0.02) measured using the HADS. The SF-36 physical component summary results were not statistically significant (F(1, 22) = 0.83, p = 0.07). The values of the SF-36 mental component summary or the 6 min walking were not statistically significant for interaction effects between time and group (F(1, 22 ) = 0.98, p = 0.59) and (F(1,22) = 0.08, p = 0.59, respectively).

**Table 2:** Baseline and 1-month physical capacity, quality of life, and anxiety and depression for the twostudy groups

**Parameter**	**Baseline**		**One month**	
	**PR (n = 12)**	**CPT (n = 12)**	**PR (n = 12)**	**CPT (n = 12)**
**Physical capacity**				
**UULEX (kg) mean(SD)**	1.13 ± 0.38	0.83 ± 0.39	1.58 ± 0.47	0.88 ± 0.38
**Endurance Testing (s) mean(SD)**	429 ± 156	503 ± 216	724 ± 202	530 ± 239
**Quality of Life**				
**SF-36 PCS (%) mean(SD)**	36 ± 8	36 ± 8	44 ± 9	37 ± 7
**SF-36MCS (%) mean(SD)**	44 ± 13	43 ± 13	49 ± 10	44 ± 11
**Anxiety and Depression**				
***HADS-A* mean(SD)**	10 ± 5	9 ± 6	5 ± 3	8 ± 5
***HADS-D* mean(SD)**	10 ± 5	10 ± 3	5 ± 3	9 ± 3
**6MWT(m) mean ± standard deviation**	425 ± 85	475 ± 86	339 ± 108	370 ± 120

SD: standard deviation; PR:pulmonary rehabilitation; CPT: chest physical therapy; UULEX: unsupported upper limb exercise test; PCS: SF-36 Physical Component Summary; MCS: SF-36 Mental Component Summary; HADS-A: Hospital Anxiety and Depression Scale - Anxiety; HADS-D: Hospital Anxiety and Depression Scale - Depression.

## Discussion

In the present study, we show that 4 weeks of PR reduced serum fibrinogen levels in patients with lung cancer and previous inflammatory respiratory disease who were eligible for lung resection. However, the change in the albumin levels of this group was not statistically significant,

As recently as 2006, the ATS and European Respiratory Society (ERS) did not mention the effect of PR on the inflammatory status of patients with LC, in contrast to studies on inflammation in COPD patients [20]. Subsequently, Jones et al. [21], using a single-group design, observed that exercise training reduced significantly the levels of intracellular adhesion molecule-1 among patients with malignant lung lesions. They also reported that aerobic training was associated with significant increases in urinary measures of oxidative status in patients with NSCLC after surgery [22]. In contrast to these previous studies, we found that serum fibrinogen levels decreased after 4 weeks of pulmonary rehabilitation. The identification of these different biomarkers of systemic inflammation among patients with malignant lung lesions is likely because of the complexity of tumor biology. Further studies are required to more clearly understand the complex relationship between exercise and tumor biology.

Vogiatzis et al. [23] studied patients with COPD without lung cancer and did not observe changes in the plasma concentrations of C-reactive protein (CRP), tumor necrosis factor-α, and interleukin-6 after a 10-week PR program. Pitsavos et al. [24] examined the association between physical activity and CRP concentration in a national sample of the United States population and observed that physical activity may reduce inflammation. Most studies on the influence of physical activity on such blood components are conducted using trained athletes or athletes undergoing training [24,25], and two population-based studies show that physical activity lowers plasma fibrinogen concentration [26,27]. A large cross-sectional study of 1284 healthy subjects, with a mean age of 55.0 ± 13.6 years, showed that fibrinogen levels decrease with exercise [28]. Similarly, the patients studied here experienced decreases in plasma fibrinogen levels.

A study of 635 patients undergoing lung resection has shown that male gender, preoperative fibrinogen level > 400 mg/dL, and duration of surgery are significantly associated with pulmonary complications [7]. Because fibrinogen levels serve to predict postoperative pulmonary complications, these data, taken together with our present findings, support the conclusion that PR is useful for lowering serum fibrinogen levels before surgery. Moreover, fibrinogen levels are influenced by other conditions, such as comorbidities [29] that may have influenced our results. However, there was a similar distribution of comorbidities in the PR and CPT groups (Table 1), which argues against a bias in the data caused by comorbidities.

There are few studies on the effects of exercise interventions designed to reduce inflammation in patients with NSCLC [21,22]. Patients studied here underwent pulmonary rehabilitation five times a week for 4 weeks. We were concerned that the 1-month delay in lung resection would affect patients’ outcomes. However, the preoperative protocols employed by the center where our research was conducted take approximately 1 month to complete as well.

There are limitations of the present research. For example, the number of subjects was small but is similar to others studies involving exercise and lung cancer. The study by Jones et al. [21] also observed a significant reduction in inflammatory biomarkers after a presurgical exercise training among 12 patients with lung cancer. Cesario et al. [30] and Bobbio et al. [31] each evaluated 12 patients with NSCLC in the preoperative period and observed an improvement in exercise capacity after pulmonary rehabilitation. Another limitation is the analysis of only one inflammatory marker. However, fibrinogen levels can be easily determined in clinical practice, and they serve as an important a predictor of postoperative pulmonary complications after lung resection [7]. There is a potential bias concerning the selection of patients with previous inflammatory respiratory comorbidities, which, along with certain therapies such as corticosteroid administration and use of bronchodilators, can influence the reduction of fibrinogen levels. However, after randomization, the two groups did not differ systematically at the outset of the study according to the presence and severity of previous pulmonary disease. Therefore, we attribute any differences in outcomes to the effects of the RP and CPT protocols. Another important limitation is the absence of follow-up of these patients.

## Conclusions

A presurgical short-term, high-intensity pulmonary rehabilitation program reduced serum fibrinogen levels in patients with NSCLC and previous inflammatory pulmonary disease. This exploratory study provides preliminary evidence to support the vigorous pursuit of future studies to investigate the role of exercise in inflammation and cancer.

## Abbreviations

LC: Lung cancer; NSCLC: Non small cell lung cancer; FEV_1_: Forced expiratory volume in the first second; FVC: Forced vital capacity; GOLD: Global initiative for obstructive lung disease; PR: Pulmonary rehabilitation; CPT: Chest physical therapy; BMI: Body mass index; PNF: Proprioceptive neuromuscular facilitation; ERS: European respiratory society; UULEX: Unsupported upper limb exercise; 6MWT: 6-minute walk test; SF-36: Short form health survey; PCS: Physical component summary; MCS: Mental component summary; HADS: Hospital anxiety and depression scale; UL: Upper limb; LL: Lower limb; ATS: American thoracic society; ICAM-1: Intracellular adhesion molecule; CRP: C- reactive protein; TNF-alpha: Tumor necrosis factor; IL-6: Interleukin-6.

## Competing interests

The authors declare that they have no competing interests.

## Authors’ contributions

EDBP contributed as the principal investigator, to the study concept and design, analysis of the results, and writing of the manuscript. MTAPM, GPFS, ASA, AGN, CMSV contributed to execution of the study and acquisition of data. RM was responsible for the statistical analysis of the data. JMdSP. and MOdMF contributed to the data analysis and interpretation and editing of the manuscript. All authors read and approved the final manuscript.

## Pre-publication history

The pre-publication history for this paper can be accessed here:

http://www.biomedcentral.com/1471-2466/14/121/prepub
